# Frequency of Spinocerebellar Ataxia type 1, 2, 3,6 and 7 and clinical profile of Spinocerebellar Ataxia type 3 in Malaysia

**DOI:** 10.1186/s40673-020-00120-2

**Published:** 2020-08-03

**Authors:** Norlinah Mohamed Ibrahim, Yue Hui Lau, Noorasyikin Ariffin, Siti Hajar Md Desa, Elena Azizan, Long Kha Chin, Shahrul Azmin Md Rani, Yusnita Yakob, Santhi Datuk Puvanarajah, Bart van de Warrenburg

**Affiliations:** 1 Neurology Unit, Department of Medicine, Universiti Kebangsaan Malaysia Medical Centre, Kuala Lumpur, Malaysia; 2 Neurology Department, Hospital Kuala Lumpur, Kuala Lumpur, Malaysia; 3 Department of Medicine, Hospital Labuan, Wilayah Persekutuan, Malaysia; 4 Molecular Laboratory, Department of Medicine, Universiti Kebangsaan Malaysia Medical Centre, Kuala Lumpur, Malaysia; 5 Molecular Genetics Department, Institute of Medical Research, Kuala Lumpur, Malaysia; 6 Department of Neurology, Donders Institute of Brain, Cognition, and Behaviour, Radboud University Medical Center, Nijmegen, Netherlands

## Abstract

Spinocerebellar ataxias (SCA) are highly heterogenous group of neurodegenerative diseases causing progressive cerebellar dysfunction. We report the first description of relative frequencies of the common SCA mutations and of phenotypic characteristics of SCA3 patients among Malaysians. Pooled data from adult Malaysian patients who had undergone genetic testing for SCA 1,2,3,6 and 7 at UKM Medical Centre and Institute for Medical Research from 2017 to 2020 were analysed. Fifteen patients with SCA 3 had detailed clinical phenotype evaluation using Inventory for Non -Ataxia Signs (INAS) and Ataxia Severity evaluation using the Scale for Assessment and Rating of Ataxia (SARA). Out of 152 adults patients who were tested for common SCA mutations, 64(42.1%) patients were tested positive for either SCA 1,2,3,6 or 7. Of the 64 positive cases, 44 (68.9%) patients were diagnosed with SCA 3 followed by SCA 2 in 13(20.3%) patients and SCA 1 in 5 (7.8%) patients. Our findings suggest that Malay race had the highest frequency of SCA (*n* = 34, 50%), followed by the Chinese (*n* = 16, 23.5%) and approximately 60 (93.8%) SCA patients had first degree family history. In conclusion, SCA 3 is the commonest SCA in Malaysia, followed by SCA 2 and SCA 1. It is important to develop a proper registry of SCA patients to further understand the true prevalence and local impact of the disease in Malaysia.

## Brief report

Spinocerebellar ataxias (SCA) are autosomal dominant, neurodegenerative disorders causing mostly adult-onset, progressive cerebellar dysfunction, with or without involvement of other central and peripheral nervous system components. To date, there are almost 50 genetic SCA subtypes worldwide [1], with SCAs 1, 2, 3, 6 and 7 being the commonest [1, 2]. The phenotypic characteristics of SCAs are heterogeneous with wide intrafamilial and interfamilial variability [3, 4]. While the prevalence of SCA is established in many Western countries [1, 3] and some East Asian countries such as China [5] and Korea [6], data on SCA in the South East Asian countries is lacking apart from two published studies from Thailand [7] and Singapore [8], there is no data on SCA in other countries within this region, possibly due to lack of testing facilities locally. Since 2017, two centres (UKM Medical Center and Institute for Medical Research (IMR), Malaysia) in Malaysia have provided genetic testing for SCA1, 2, 3, 6 and 7, which enabled identification of cases with one of these more common SCA subtypes. UKM Medical Center is a tertiary referral center, which provides diagnosis and clinical management of SCA in Malaysia, whereas IMR is a laboratory testing facility which receives patient samples for SCA genetic testing from other centers throughout Malaysia. Malaysia is a multi-ethnic country with a population of 32.6 million (2019), of which 3.2 million are non-citizens. The major ethnic group is Malay (69.3%) followed by Chinese (22.8%), Indians (6.9%) and natives (1%). Here, we report the first description of relative frequencies of the common SCA mutations and of phenotypic characteristics of SCA3 patients among Malaysian adults.

The frequency analysis was conducted as part of audit review using data of Malaysian patients, aged 18 and above who had undergone genetic testing for SCA1, 2, 3, 6 and 7 at UKM Medical Centre, Kuala Lumpur and Institute for Medical Research, Kuala Lumpur from February 2017 to February 2020. Non-Malaysian patients who had tested positive were excluded. Data on age, race, family history and age of testing were available for analysis. Genetic analysis was performed in UKMMC and IMR using both standard PCR and triplet-primed PCR techniques respectively.

Clinical profile evaluation for SCA 3 using the Inventory for Non-Ataxia Signs (INAS) [9] and the Scale for Assessment and Rating of Ataxia (SARA) [10] were available separately for fifteen SCA 3 patients who were enrolled in another clinical study on SCA 3 in UKM Medical Center [11]. Ethical approval was obtained from the institution’s Ethics Committee (FF-2018-082).

Out of 152 patients who had undergone genetic testing from 2017 to 2020, 64 (42.1%) patients tested positive for either SCA1, 2, 3 or 6. Of these 64 positive cases, SCA3 was the commonest, detected in 44 (68.9%) patients, followed by SCA2 in 13 (20.3%) and SCA1 in 5 (7.8%) and SCA6 in 2(3%). None of the patients were tested positive for SCA 7 (Table 1). A positive family history in a first degree relative was present in 60 out 64 patients (93.8%), while 3 (4%) cases were sporadic, and for the remaining 7 patients’ data on family history were incomplete. SCA3 affected all races including Malays. There was no ethnic preponderance for any of the SCAs tested. Consistent with the ethnic composition of the Malaysian population, the majority of SCA mutation carriers were Malay (*n* = 34, 50%), followed by Chinese (*n* = 16, 23.5%) (Table 1).

**Table 1:** Demographics of patients with Spinocerebellar Ataxia Type 1, 2, 3, 6 and 7 in Malaysia

Type of SCAs (n, %)	SCA 1 5(7.8%)	SCA 2 13 (20.3%)	SCA 3 44 (68.9%)	SCA 6 2 (3%)	SCA 7 0 (0)
Gender (n, %)					
Male	2 (3)	9 (14.2)	22 (34.4)	2 (3)	0 (0)
Female	3 (4.7)	4 (6.3)	22 (34.4)	0 (0)	0 (0)
Ethnicity (n, %)					
Malay	0 (0)	10 (15.6)	24 (37.5)	0 (0)	0 (0)
Chinese	1 (1.6)	1 (1.6)	13 (20.3)	2 (3)	0 (0)
Indian	3 (4.7)	1 (1.6)	3 (4.7)	0 (0)	0 (0)
Punjabi	0 (0)	0 (0)	4 (6.3)	0 (0)	0 (0)
Others	1 (1.6)	1 (1.6)	0(0)	0 (0)	0 (0)
Family history (n, %)					
Yes	4 (6.3)	11 (17)	44 (68.9)	1(1.6)	–
No	–	1 (1.6)	–	1(1.6)	–
Unknown	1 (1.6)	1 (1.6)	–	–	–

In 15 patients with SCA3 whose clinical profile was evaluated, the mean age of onset was 31.2 ± 8.1 years and the mean age of presentation was 35.6 ± 9.2 years. The mean SARA score was 12.3 ± 8.9. The CAG repeats expansion ranged, 68 to 83, with a mean CAG repeat expansion of 74 ± 4. A cerebellar syndrome was present in 100% of the cases, pyramidal signs in 60%, parkinsonism in 13%, and dystonia in 13%. Ocular manifestations such as nystagmus, ophthalmoplegia, and saccadic abnormalities were common in SCA3 patients, affecting up to 50% of patients. Detailed phenotypic characteristics based on INAS are presented in Table 2.

**Table 2:** Clinical and phenotypic characteristics of SCA 3 patients in Malaysia using INAS

Baseline characteristics (*n* = 15)	
Gender:	
Male (n, %)	9.0(60.0%)
Female (n, %)	6.0(40.0%)
Mean age of presentation (years,(SD))	35.6(±9.2)
Mean age of onset (years, (SD)	31.2(±8.1)
Mean duration of illness (years, (SD))	4.5(±2.9)
Median time to wheel chair (months, (IQR))	12.0(12.0–24.0)
Median time to ophthalmoplegia (months, IQR))	12.0(6.0–21.0)
Mean SARA at baseline score (score (SD))	12.3(±8.9)
Phenotypic Characteristics (n, %)	
Broken smooth pursuit	7(46.7)
Square jerk fixation	7(46.7)
Downbeat nystagmus	4(26.7)
Gaze evoked horizontal nystagmus	8(53.3.)
Gaze evoked vertical nystagmus	3(20.0)
Ophthalmoparesis on horizontal nystagmus	7(46.7)
Opthalmoparesis vertical gaze	7(46.7)
Slowing saccades	7(46.7)
Hypermetric saccades	3(20.0)
Hypometric saccades	7(46.7)
Diplopia	7(46.7)
Ophthalmoplegia	7(46.7)
Hyperreflexia	9(60.0)
Extensor plantar reflex	3(20.0)
Clonus	2(13.3)
Spasticity	6(40.0)
Rigidity	2(13.3)
Resting tremor	0 (0)
Dystonia	2(13.3)
Paresis	0 (0)
Muscle atrophy	2(13.3)
Fasciculation	2(13.3)
Chorea	0 (0)
Impaired vibration sense	1(6.7)
Dysarthria	14(93.3)
Dysphagia	3(20.0)
Cognitive impairment	1(6.7)

Similar to worldwide data, SCA3 was the commonest SCA in Malaysia, followed by SCA2 and SCA1, whereas SCA6 and SCA7 were the least common. Except for Korea [6] and India [12] where SCA2 was the commonest, other Asian countries have reported SCA3 to be the commonest. A study conducted in Thailand also reported that SCA3 was the commonest, followed by SCA1, SCA2 and SCA6 [7]. Similar to our findings, a Singapore study reported that SCA3 was the commonest, followed by SCA2. They also reported that SCA2 affected the Malay ethnic group exclusively [8]. However, our data showed that SCA2 affected all races and did not affect the Malay ethnic group exclusively, despite a similar ethnic composition to Singapore. Apart from these two studies [7, 8] there is no published data on the prevalence of SCA in other South East Asian countries. As expected, there was a high diagnostic yield of 93.8% in patients with a positive family history, but we also identified sporadic presentations of SCAs. The clinical phenotype of patients with SCA3 was similar to that reported in other countries [3, 4], with a predominant cerebellar syndrome with prominent pyramidal signs in 60% and extrapyramidal signs in 26%. One important limitation of this study is that we were unable to account for those who had their genetic testing done elsewhere, which could have led some inaccuracies in the relative frequencies calculated. Still, this study is the first to report the frequency of common SCAs in Malaysia, which could be used as a stepping stone in conducting a large-scale epidemiological study on SCA in Malaysia. We believe the availability and the relatively lower cost of genetic testing locally had increased the number of tests conducted. As data on SCA in the South East Asian regions is still lacking, there is a need for collaborative efforts among experts to look into the challenges and priorities in the detection and management of SCA in this region. Facilitating diagnosis, with a proper registry are key in enhancing the care and comprehensive management of SCA patients within this region, and to include patients in future trials.

## Data Availability

The datasets used and/or analysed during the current study are available from the corresponding author on reasonable request.
